# Supplementary material for: Complementary Roles of GCN5 and PCAF in Foxp3+ T-Regulatory Cells
Source: Cancers (Basel). 2019 Apr 18;11(4):554. doi: 10.3390/cancers11040554 (PMC6520961; doi:10.3390/cancers11040554)
Supplement: Supplementary file 1 [file cancers-11-00554-s001.pdf]

Fig. S1: T cell deletion of GCN5 in Foxp3+ Tregs

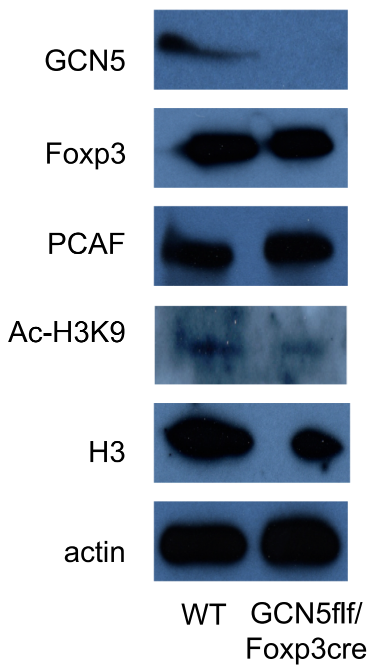

Fig. S1A: Treg-specific GCN5 deletion decreased AcH3K9 but not Foxp3 levels; data are representative of results in 4 mice/group at 6 weeks of age.

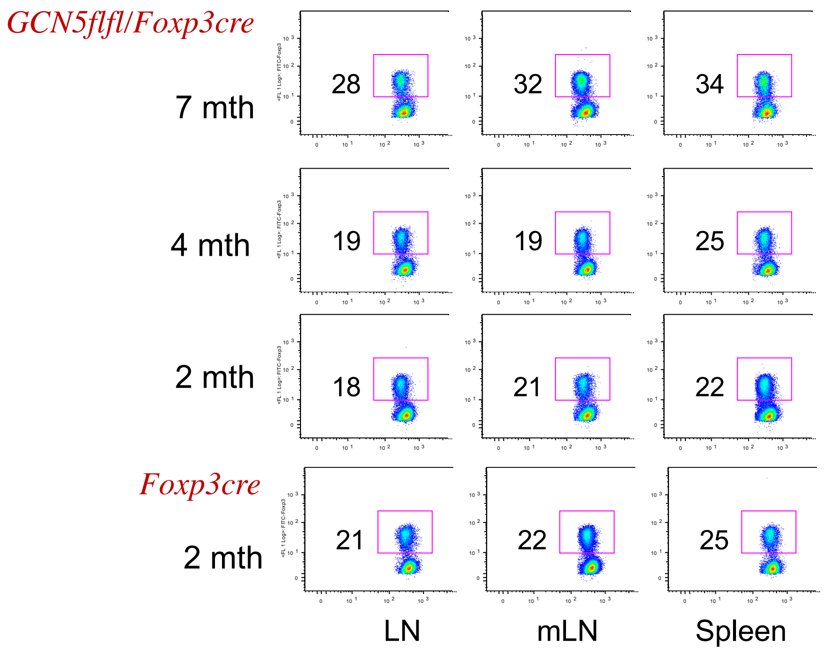

Fig. S1B: Flow cytometric evaluation of Foxp3+CD4 T cells by age in peripheral lymph nodes (LN), mesenteric LN (mLN), and spleen; data are representative of results in 4 mice/group at the ages shown.

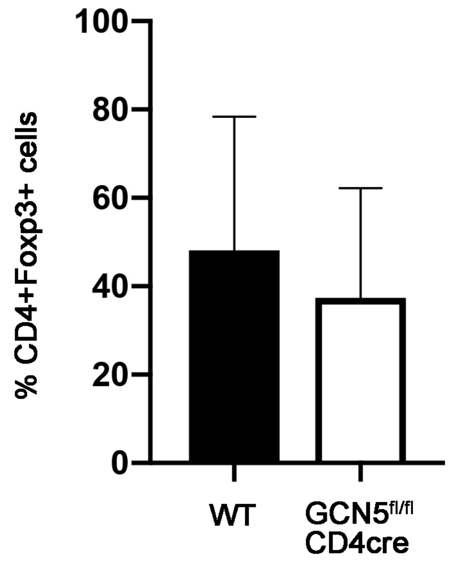

Fig. S1C: No significant difference in iTreg development from WT T cells vs. T cells lacking GCN5 expression (mean ± SD, 3 experiments).

Fig. S2: T cell deletion of GCN5 in all T cells (using CD4^{cre})

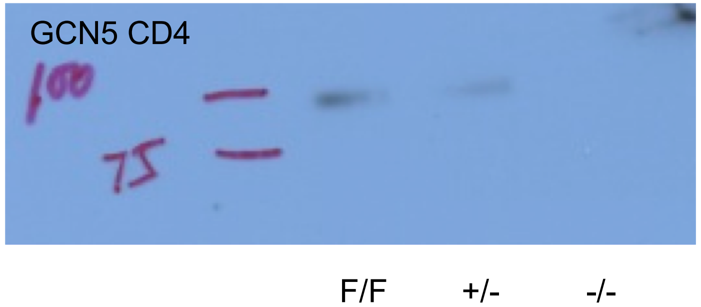

Fig. S2A: Western blots of T cells for GCN5 expression.

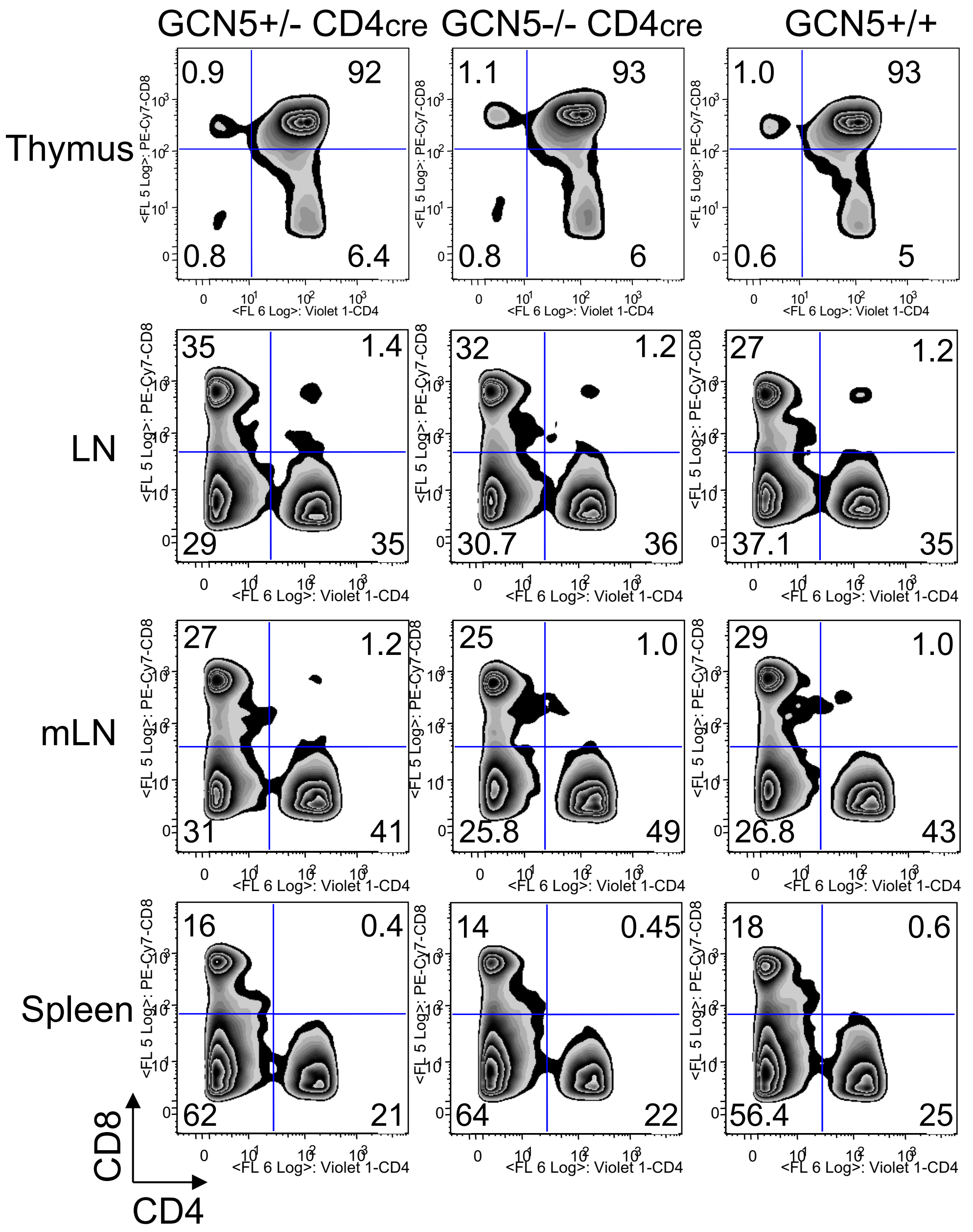

Fig. S2B: Flow cytometric evaluation of the effects of GCN5 deletion in T cells obtained from the thymus, peripheral lymph nodes (LN), mesenteric LN (mLN), and spleen; data are representative of results in 4 mice/group.

Fig. S3: T cell deletion of GCN5, using CD4^{cre}, did not affect the level of T cell activation under resting conditions

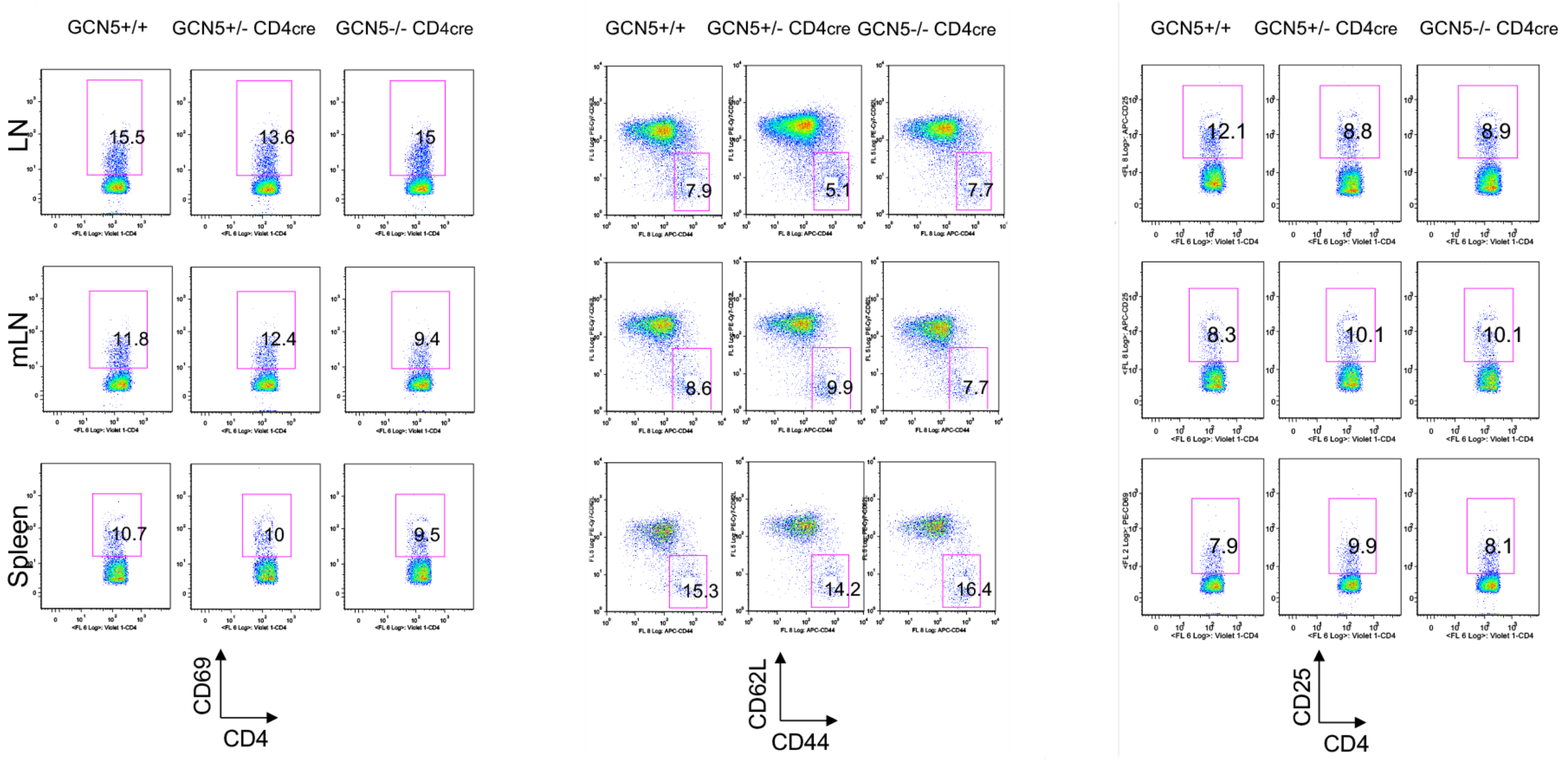

Fig. S3: Flow cytometric evaluation of the expression of T cell activation markers within peripheral LN, mesenteric LN (mLN) or spleen; data are representative of results in 4 mice/group.

Fig. S4: T cell deletion of GCN5 impaired CD4+CD25- Teff proliferation

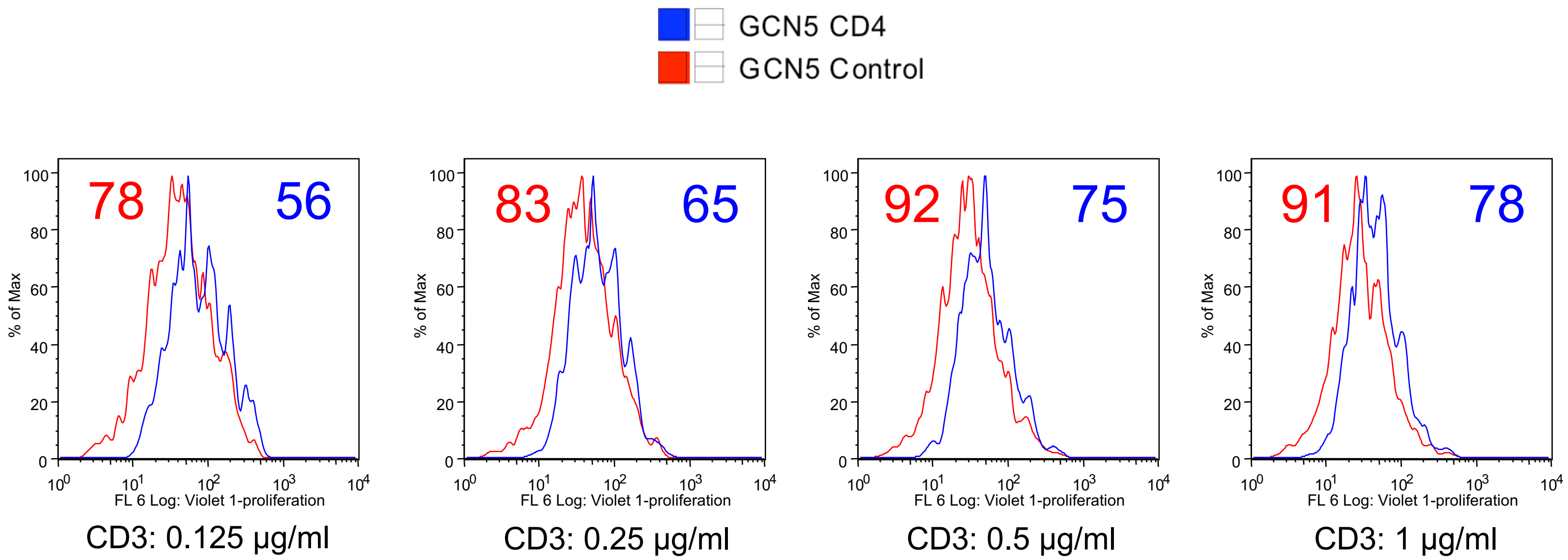

Fig. S4: GCN5 deletion decreased CD3 mAb-induced T cell proliferation *in vitro* ($p<0.05$); data are representative of 3 experiments.

Fig. S5: GCN5 deletion impairs CD4+ T cell production of IL-2, and in WT cells GCN5 promotes acetylation of p65/NFκB and histone-H3 and is recruited to the IL-2 promoter during T cell activation

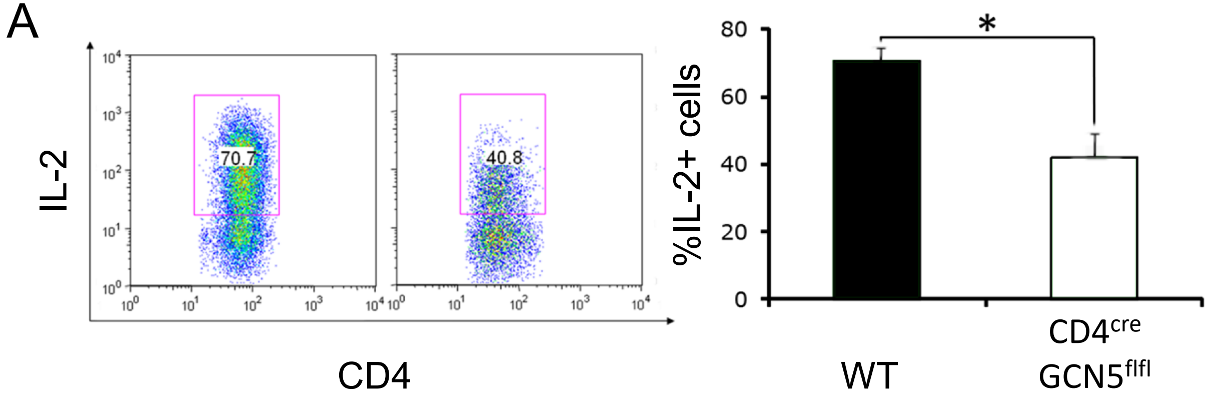

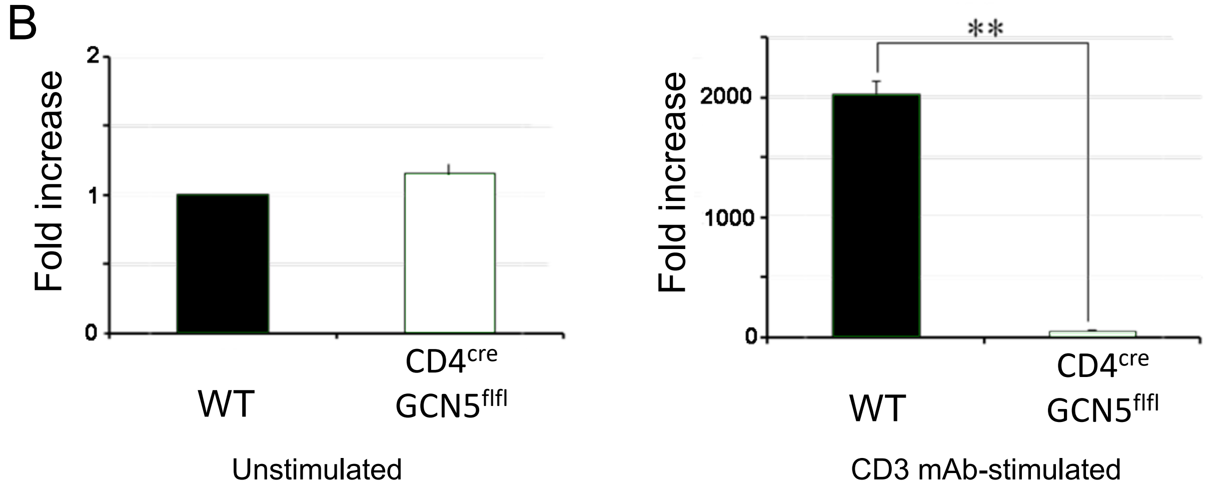

Fig. S5A: Flow cytometric evaluation of intracellular IL-2 production (representative of 3 /group) using T cells activated *in vitro* using CD3 mAb (24 h) followed by PMA (3 ng/ml) and ionomycin (1 μM) plus Golgistop (0.5 μM). Data representative from 3 experiments shown at left, with overall data at right (*P<0.05).

Fig. S5B: IL-2 mRNA expression (qPCR) by unstimulated CD4 T cells (left) or cells stimulated for 24 h with CD3 mAb (right); 4 samples/group, **p<0.01).

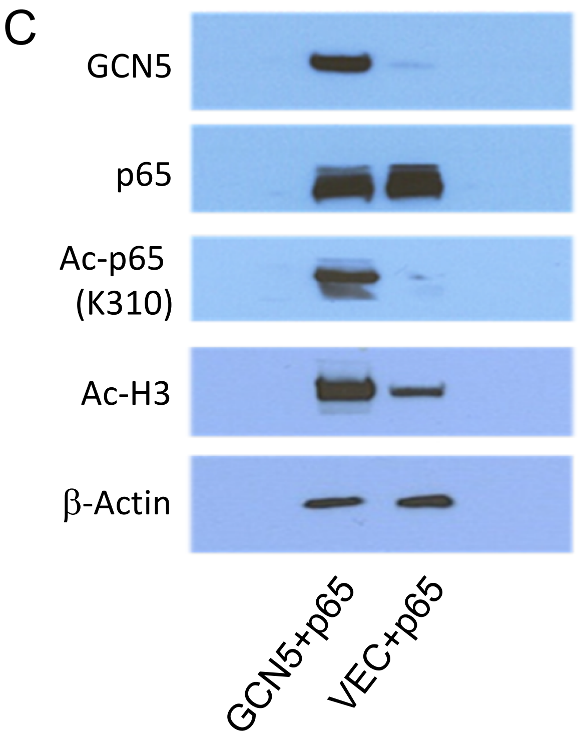

Fig. S5C: Western blots of nuclear extracts of 293 cells transfected with GCN5 plus p65, or empty vector (VEC) plus p65.

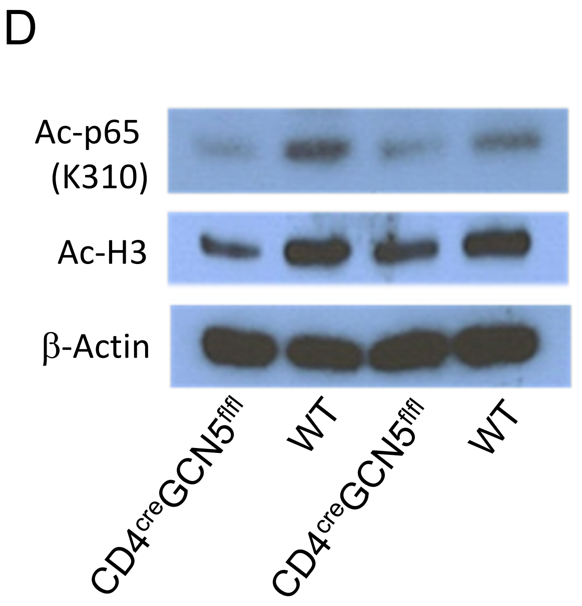

Fig. S5D: Western blots of nuclear extracts of CD4+ T cells cells stimulated with CD3 mAb for 4 h.

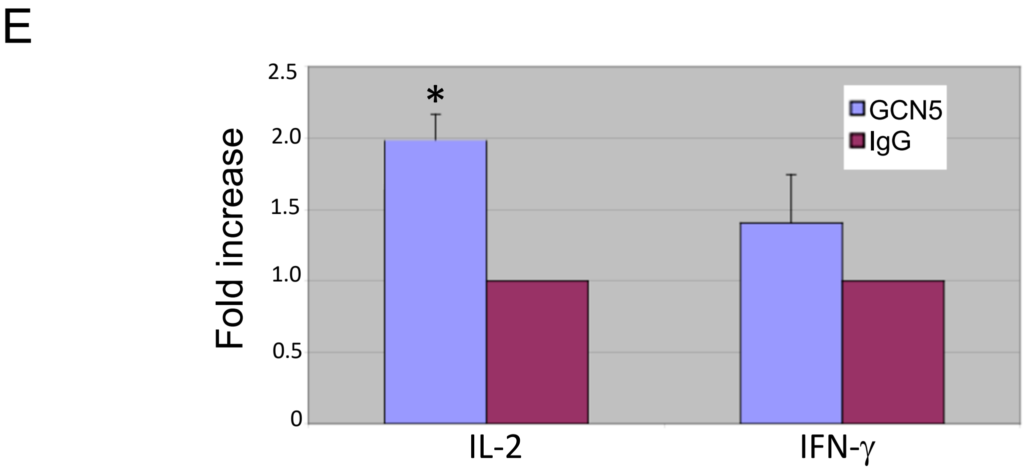

Fig. S5E: ChIP analysis of GCN5 recruitment to the IL-2 and IFN-g promoters of CD4 T cells stimulated with CD3 mAb for 4 h, using 3 samples/group, **p<0.05).

Fig. S6: T cell deletion of GCN5 promotes cardiac allograft survival

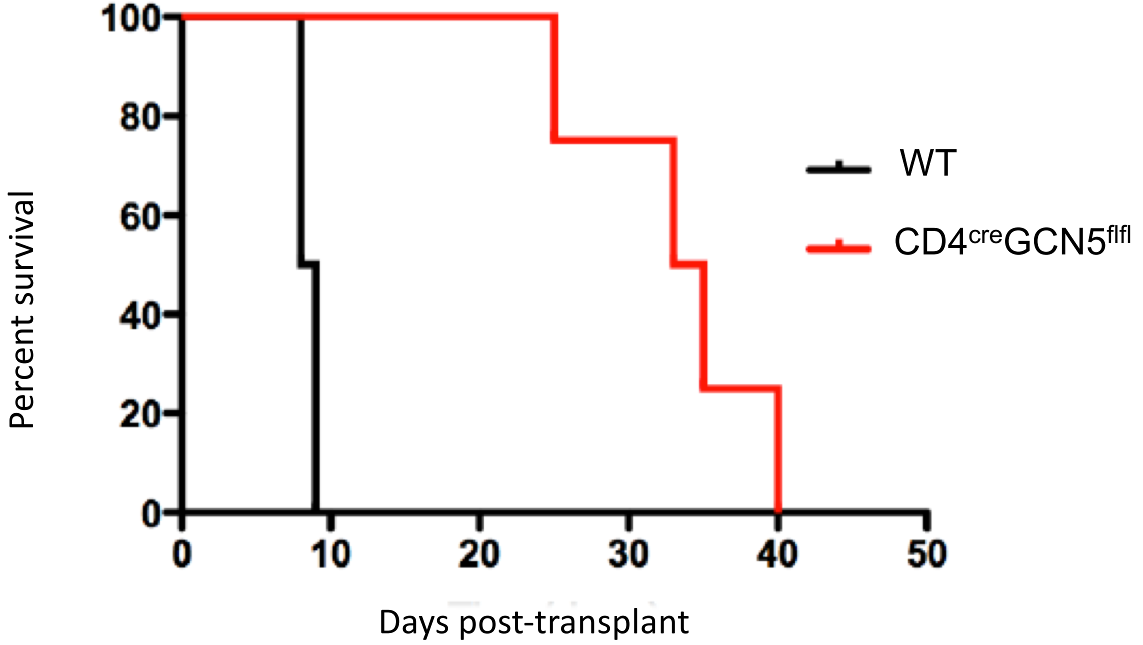

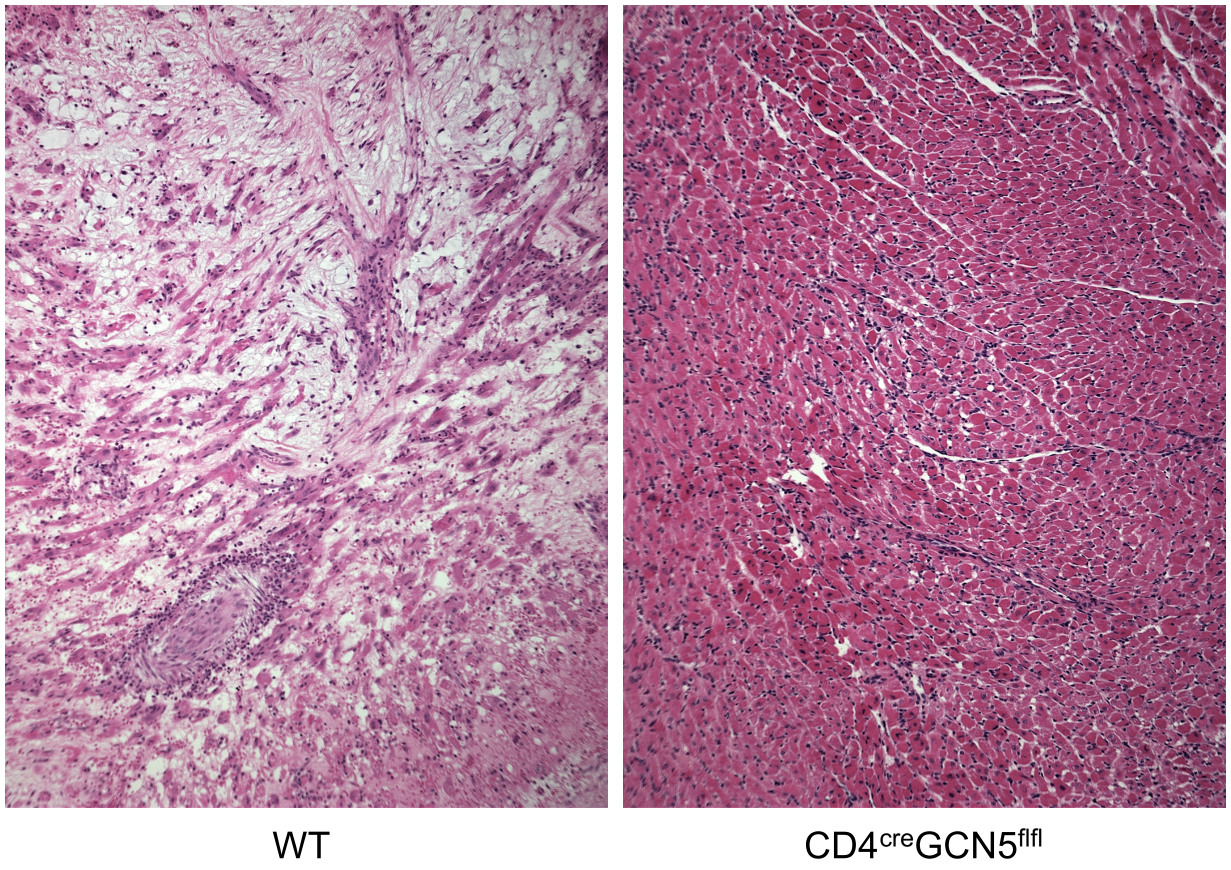

Fig. S6A: Survival curves after cardiac transplantation (BALB/c->C57BL/6) using 4 mice/group. Allograft recipients, all of which received 14 d of low dose rapamycin (0.1 mg/kg/d) from the day of transplantation, were either WT or lacking GCN5 expression within their T cells; log-rank (Mantel-Cox) test, $p = 0.0084$.

Fig. 6B: H&E-stained paraffin sections of cardiac allografts harvested from WT or CD4^{cre}GCN5^{flfl} recipients at day 9 post-transplant; histology is representative of 4 mice/group and original magnifications x125.

Fig. S7: Cumulative data (mean ± SD) from 3 experiments and relating to Figures 2F, 2G and 3A

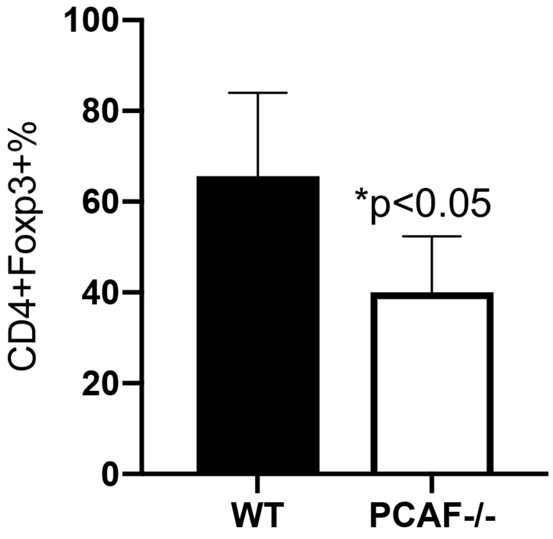

Cumulative data on Treg numbers after CD3/CD28 activation, relevant to Figure 2F

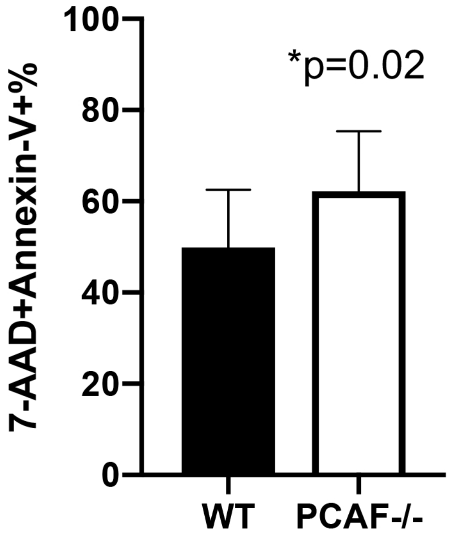

Cumulative data on Treg apoptosis after CD3/CD28 activation, relevant to Figure 2G

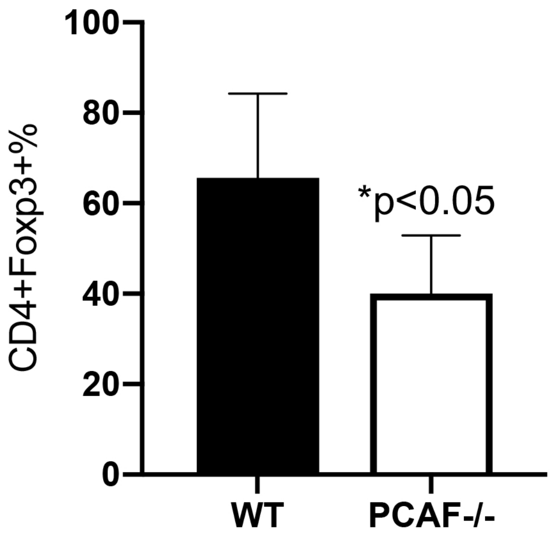

Cumulative data of iTreg development, relevant to Figure 3A

Fig. S8: PCAF deletion did not impair IFN-γ production by TH1 cells

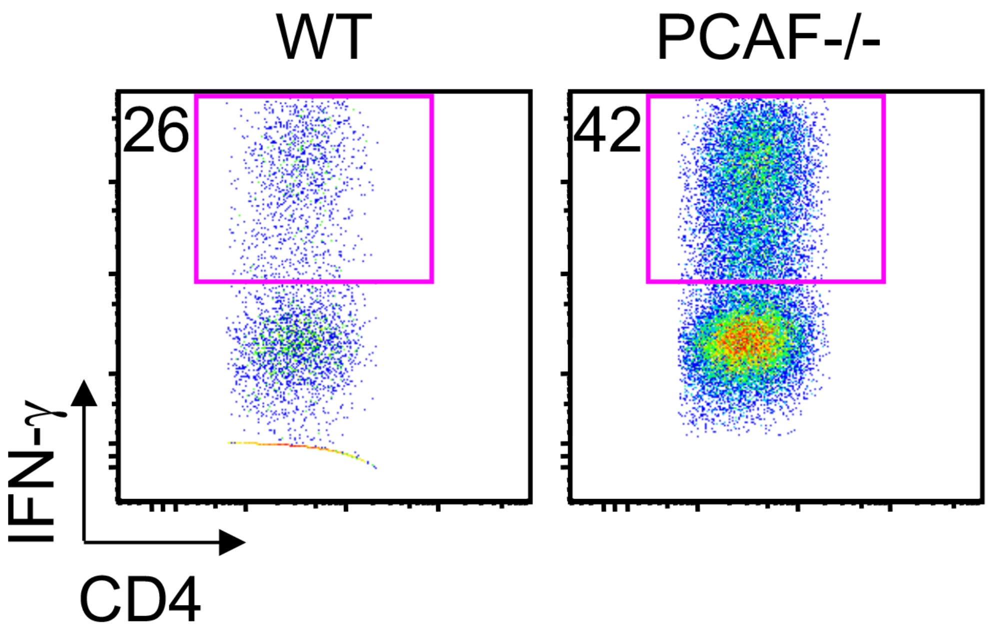

Fig. S7: Naïve CD4+CD25-CD62L^{hi}CD44^{lo} T cells sorted from PCAF-/- or WT mice were cultured under Th1 skewing conditions for 4 days. Intracellular staining showed increased IFN-γ production in PCAF-/- vs. WT CD4+ T cells.

Fig. S9: Cumulative data (mean ± SD) from 3 experiments and relating to Figures 5E and 5F

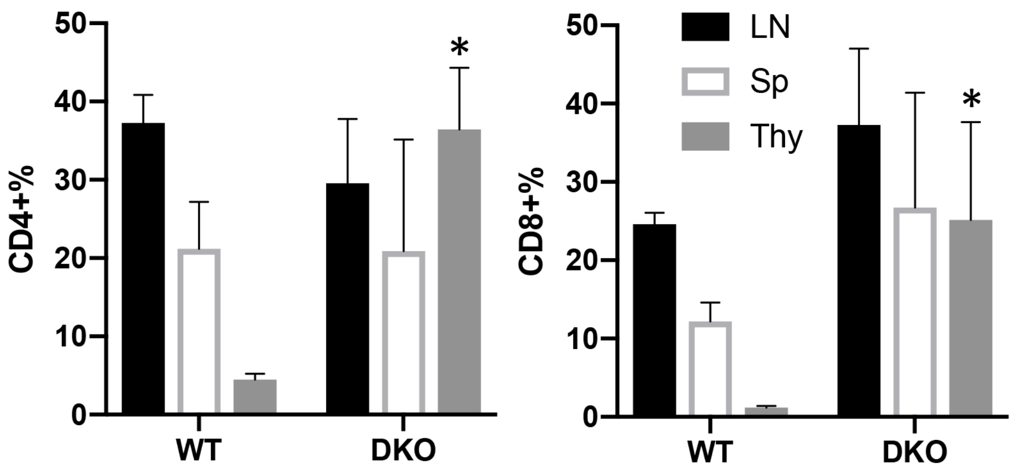

Cumulative data of CD4 and CD8 T cells, relevant to Figure 5E, *p<0.05

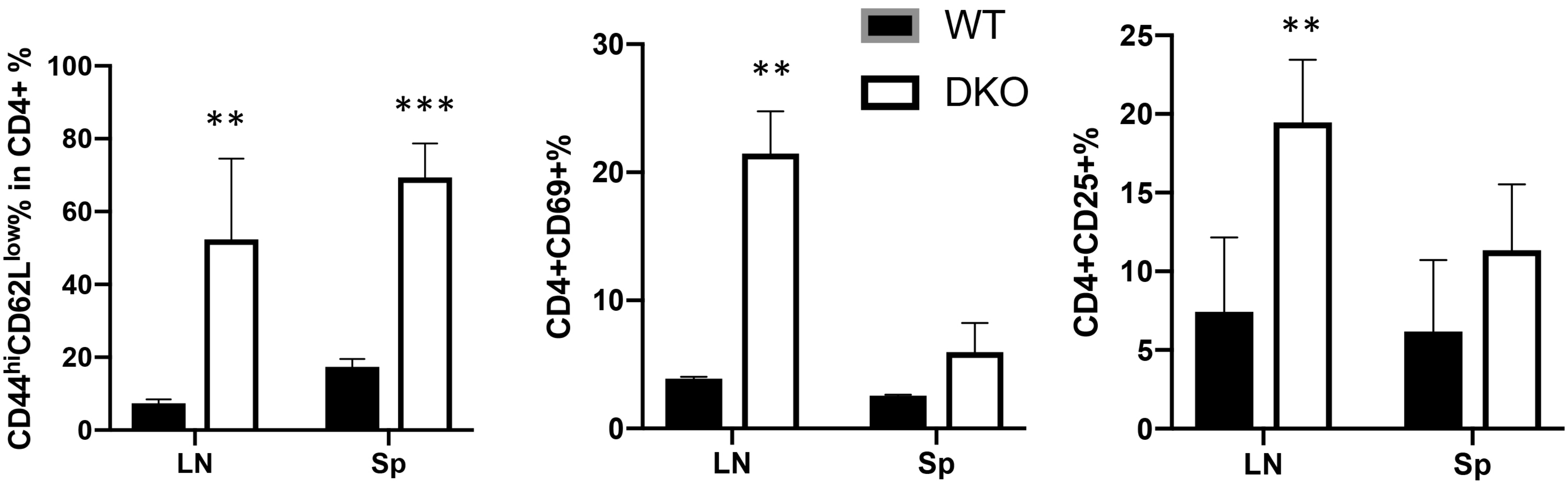

Cumulative data of CD4 T cell activation relevant to Figure 5F, **p<0.01, ***p<0.001

Fig. S10: Cumulative data (mean ± SD) from 3 experiments and relating to Figures 5G and 5H

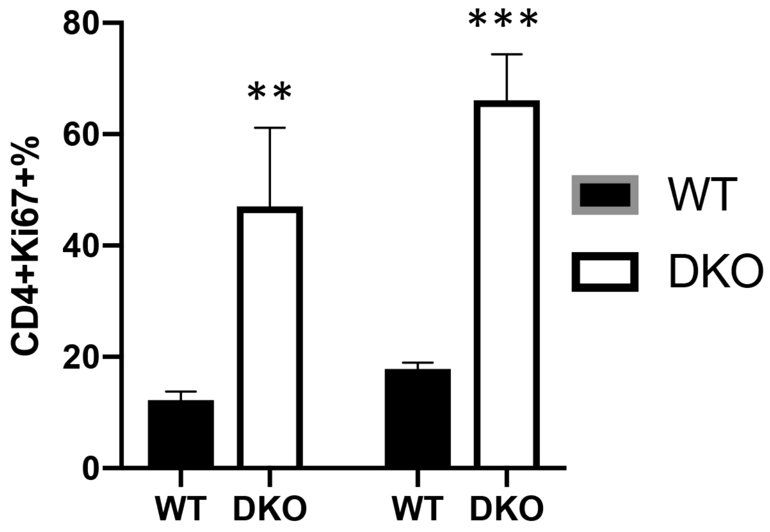

Cumulative data of CD4 proliferation relevant to Figure 5G, **p<0.01 and ***p<0.001

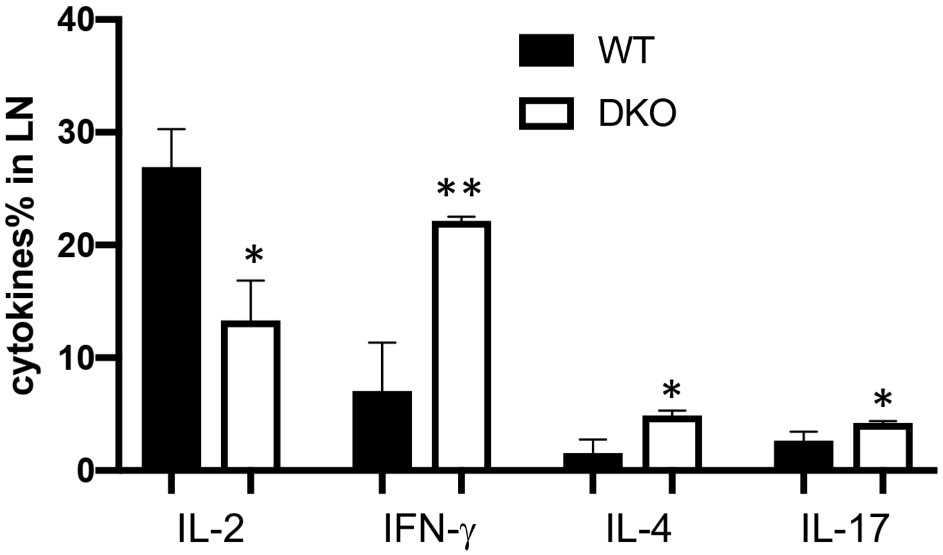

Cumulative data of T cell cytokine production relevant to Figure 5H, *p<0.05, **p<0.01

Fig. S11: Cumulative data (mean ± SD) from 3 experiments and relating to Figure 6A

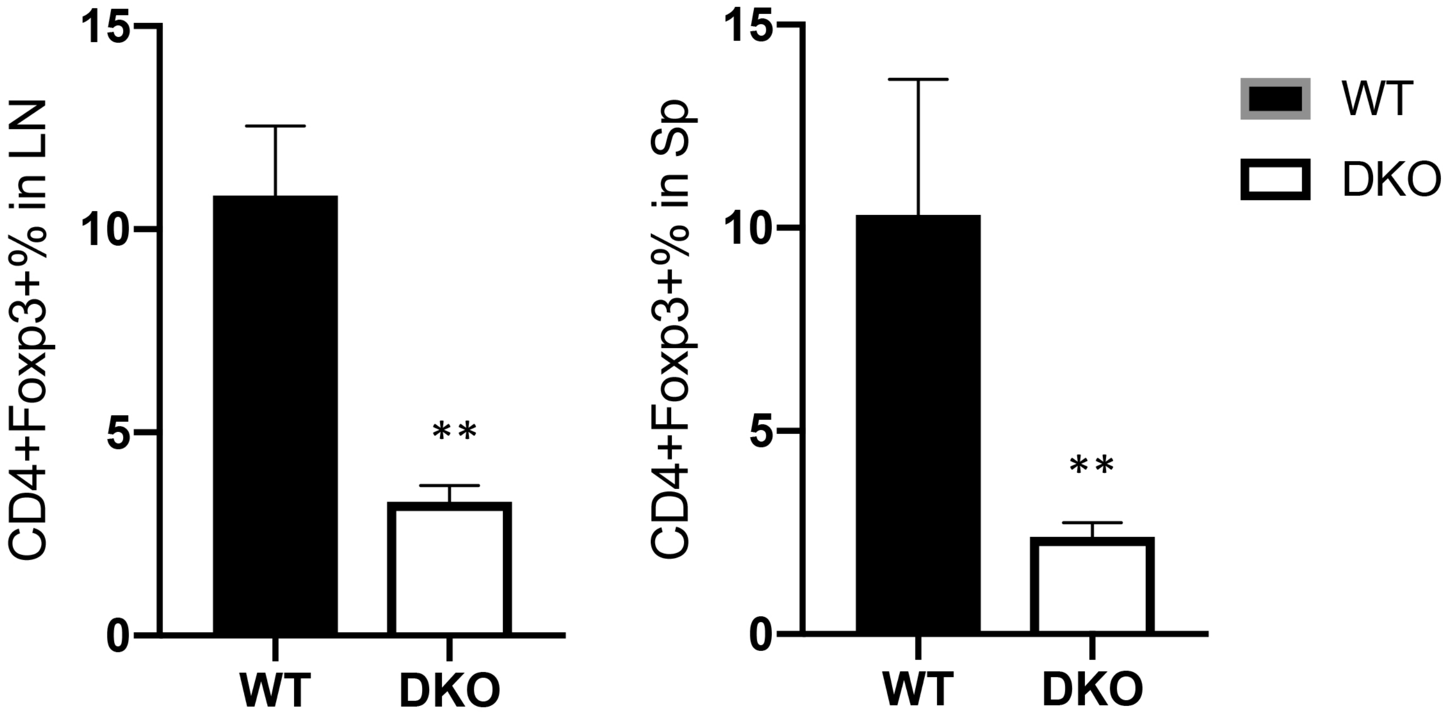

Cumulative data of Foxp3+ Treg percentages in LNs and spleens from DKO vs. WT mice, relevant to Figure 6A, **p<0.01
